# An Optimized Editing Approach for Wheat Genes by Improving sgRNA Design and Transformation Strategies

**DOI:** 10.3390/ijms26083796

**Published:** 2025-04-17

**Authors:** Rui-Xiang Zhang, Yun-Fei Zhang, Hao Yang, Xiao-Dong Zhang, Zheng-Guang Yang, Bin-Bin Li, Wei-Hang Sun, Zi Yang, Wen-Ting Liu, Kun-Ming Chen

**Affiliations:** 1 State Key Laboratory for Crop Stress Resistance and High-Efficiency Production, College of Life Sciences, Northwest A&F University, Xianyang 712100, China; zhangruixiang@nwafu.edu.cn (R.-X.Z.); zyf03200329@nwafu.edu.cn (Y.-F.Z.);; 2 Key Laboratory of Agro-Products Quality and Safety of Xinjiang, Laboratory of Quality and Safety Risk Assessment for Agri-Products (Urumqi), Key Laboratory of Functional Nutrition and Health of Characteristic Agricultural Products in Desert Oasis Ecological Region (Co-Construction by Ministry and Province), Ministry of Agriculture and Rural Affairs, Institute of Quality Standards & Testing Technology for Agri-Products, Xinjiang Academy of Agricultural Sciences, Urumqi 830091, China; 3 College of Natural & Agricultural Sciences, University of California, Riverside, CA 92507, USA; zyang019@ucr.edu

**Keywords:** genome editing, wheat mutant creation, sgRNA selection, transformation strategies, BSMV

## Abstract

Hexaploid wheat has a large genome, making it difficult for transgenes to produce phenotypes due to gene redundancy and tight linkage among genes. Multiple gene copies typically necessitate multiple targeting events during gene editing, followed by several generations of self-crossing to achieve homozygous genotypes. The high cost of transgenesis in wheat is another issue, which hinders the easy availability of gene-edited materials in wheat. In this study, we developed a comprehensive approach to improve wheat gene editing efficiency. First, we established a protoplast-based system to evaluate the relative efficiency of gene editing targets, which enabled the rapid and effective selection of optimal sgRNAs. We then compared two transformation strategies: biolistic bombardment and Agrobacterium-mediated transformation for generating edited wheat lines. Although biolistic bombardment showed higher initial editing efficiency, Agrobacterium-mediated transformation proved more effective for obtaining homozygous mutants. Notably, we discovered that deploying the same sgRNA through different vectors enhanced editing efficiency, whereas overlapping but distinct sgRNAs exhibited interference effects. Finally, we optimized the VITF-edit (virus-induced transgene free editing) technique using BSMV delivery to establish a relatively simple and easily applied wheat gene editing method for general laboratories.

## 1. Introduction

Wheat (*Triticum aestivum* L.), a hexaploid species with AABBDD genomes, represents one of the world’s three major cereal crops and contributes approximately 20% of the global daily caloric intake. The increasing global population has intensified focus on wheat production and its economic significance, particularly regarding genetic improvements for enhanced yield and quality. As commonly understood, differences in gene function are a hallmark of evolution [1], and they are also the fundamental source of excellent agronomic traits. The advent of gene editing technologies has revolutionized traditional breeding approaches by enabling precise genetic modifications, superseding conventional random mutagenesis methods for creating novel alleles [2]. Among these, CRISPR-based gene editing systems have emerged as particularly powerful tools due to their versatility in vector construction and high knockout efficiency, establishing themselves as cornerstone technologies in modern molecular breeding programs [3,4].

Recent advances in gene editing technologies have substantially accelerated progress in wheat breeding and genetic research. Recently, the *TaDUO-B1* gene, which can significantly increase wheat yield, was reported. Deletion of this gene has been found to alter the development of wheat inflorescences, resulting in the formation of a higher number of spikelets [5]. Similarly, the development of the *Tamlo-R32* mutant line, featuring a precise 304 kb deletion at the *MLO-B1* locus, demonstrates the capability to engineer powdery mildew resistance while maintaining normal growth and yield parameters [6]. Further exemplifying the versatility of CRISPR-Cas9 systems, the targeted knockout of *TaPsIPK1* has conferred broad-spectrum resistance to stripe rust without compromising essential agronomic traits [7]. The application scope of gene editing tools in wheat has expanded beyond conventional gene knockouts. For instance, CRISPR-based cytosine base editors (CBEs) have enabled precise single-nucleotide modifications in genes such as *TaMTL* and *TaALS*, which regulate haploid formation and herbicide resistance, respectively [8]. The increasing understanding of single nucleotide polymorphisms (SNPs) and their phenotypic associations has revealed that Base Editors (BEs) can generate desirable agronomic traits without fitness costs [9,10,11,12], positioning SNP-based breeding through BEs as a promising approach for accelerating crop improvement [13,14].

Despite these advances, significant challenges persist in wheat genome editing, primarily stemming from its complex hexaploid genome architecture (AABBDD) and substantial genome size (16 Gbp) [15]. These inherent characteristics frequently impede the manifestation of edited phenotypes following genetic transformation and complicate target gene analysis. Gene linkage effects further constrain progress in many instances [16], contributing to a notable lag in wheat genome editing capabilities compared to other cereal crops [17]. Although current transgenic technologies have demonstrated success in select wheat germplasm [18], their large-scale commercial application remains constrained by genotype-dependent limitations. The transformation efficiency of wheat varies greatly among different genotypes, with reported transformation rates ranging from 1.5% to 51% across multiple wheat genotypes [19]. The occurrence of gene-editing events is not only closely related to the transformation efficiency of the gene-editing vector but is also influenced by a variety of factors, including the quality of sgRNA design, the location of the target gene, and the gene copy number. Additionally, due to the hexaploid nature of wheat, it is particularly challenging to generate editing events simultaneously in the A, B, and D genomes. The low transformation efficiency and slow rate of mutant homozygosity further complicate the quantification and statistical analysis of gene-editing frequencies in wheat [20]. Recent efforts to overcome these barriers have yielded promising results, such as the enhancement of regeneration capacity and editing efficiency through *TaWOX5* expression cassettes [21]. Additionally, the implementation of wheat-specific promoters, particularly *TaU6* and *TaU3*, has improved gene editing efficiency [22,23]. However, the substantial costs associated with both Agrobacterium-mediated and biolistic bombardment transformation methods, compared to those for other plant species, create accessibility barriers for many research laboratories. This economic constraint particularly affects general research facilities, limiting their ability to conduct comprehensive gene function analyses. Consequently, these technical and economic challenges significantly restrict the integration of multiple beneficial agronomic traits within individual wheat varieties, with the creation of gene-edited mutants remaining a formidable challenge for many laboratories, ultimately impeding progress in wheat breeding programs.

This study explored a relatively easy-to-implement, high-throughput wheat gene editing system to make the technology more accessible in general laboratories. Through systematic investigation, we first established a protoplast-based system to evaluate sgRNA editing efficiencies, providing rapid assessment of target site effectiveness. Subsequently, we implemented a multi-gene editing strategy that successfully generated homozygous knockout lines of *TaNADK3* and *TaCML72-7D* genes within abbreviated timeframes, achieving homozygous mutation rates of 2.33% (2/86) and 44.44% (24/54) in the T_0_ generation, respectively. A comparative analysis of transformation methods revealed that although biolistic bombardment demonstrated higher initial editing efficiency, Agrobacterium-mediated transformation facilitated more reliable homozygous mutant generation. Notably, we discovered that repeated transformation with identical sgRNA sequences enhanced mutation efficiency, whereas overlapping but non-identical sgRNA sequences exhibited interference effects that reduced editing efficiency. Furthermore, we optimized the VITF-edit (virus-induced trans-gene-free editing technique) system based on BSMV delivery, establishing refined technical parameters. These collective strategies represent a significant advancement toward making CRISPR-based wheat gene editing more accessible for general research laboratories.

## 2. Results

### 2.1. Evaluation of sgRNA Editing Efficiency in Wheat Protoplasts

**Figure 1:**
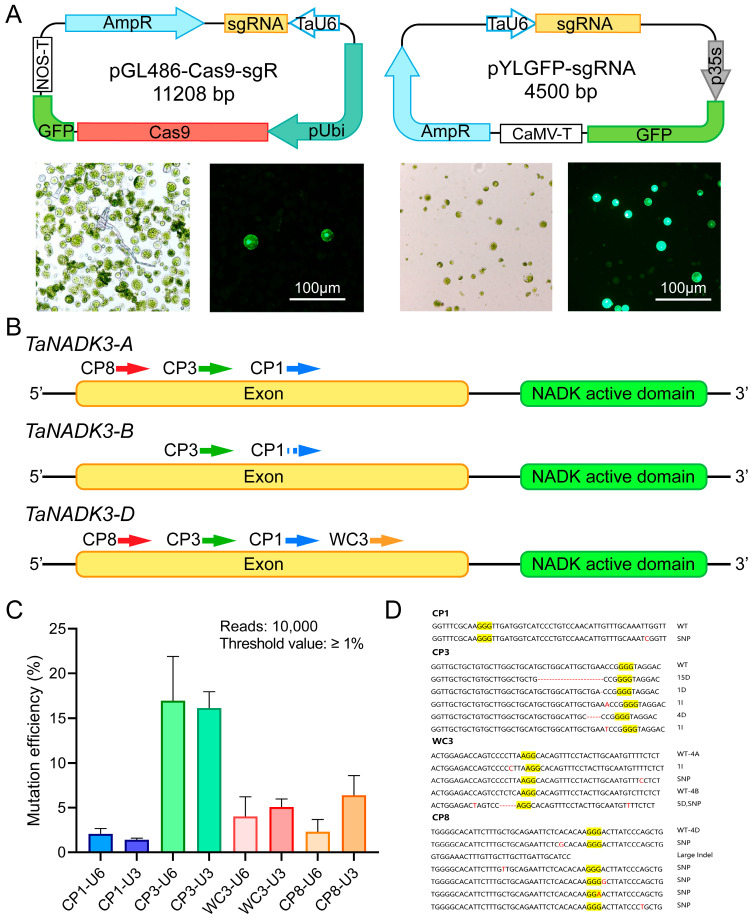
Establishment of the wheat protoplast knockout system. (**A**) Transformation effects obtained by transforming wheat protoplasts with pGL486-Cas9 and pYLGFP. (**B**) Design of *TaNADK3* sgRNA targets, respectively. The bar in the figure represents 100 μm. (**C**) Statistical gene editing efficiency of four sgRNAs targeting protoplasts at Hi-TOM 10,000 reads. (**D**) Main types of mutations generated by different targets. (Yellow highlighted part is the PAM sequence, and red indicates the type of editing).

To establish an efficient gene editing system in wheat, we developed and compared two vector systems for protoplast transformation: pGL486-Cas9 (11,208 bp) and pYLGFP (4500 bp), both utilizing the *TaU6* promoter to drive sgRNA expression (Figure 1A). We selected the *TaNADK3* gene in wheat as our editing target, which encodes a chloroplast-localized kinase capable of converting NAD^+^ to NADP^+^. Mutations in its homologous gene, *AtNADK2*, in Arabidopsis thaliana result in retarded plant growth and developmental delay [24].

We employed two sgRNA design websites, CRISPR-Cereal (abbrev. CP) and WheatCrispr (abbrev. WC), to predict potential sgRNA targets for *TaNADK3*. Based on the prediction results, we selected four sgRNAs targeting different regions of *TaNADK3* and named them CP1, CP3, WC3, and CP8 according to their source websites and ranking (Figure 1B, Table S1, Figure S1). These constructs were transformed into protoplasts isolated from Bobwhite-Cas9^+^ wheat. Comparative analysis revealed that pYLGFP-sgRNA exhibited significantly higher transformation efficiency than pGL486-Cas9-sgRNA, indicating an inverse correlation between vector size and transformation success in wheat protoplasts.

Subsequent Hi-TOM sequencing analysis at 10,000 read depth demonstrated differential editing efficiencies among the four sgRNAs. The CP3 target achieved the highest editing efficiency, suggesting that the simultaneous targeting of all three homologous copies (A, B, and D genomes) enhanced overall editing effectiveness. Additionally, CP8, which targeted two homeologous copies (4A and 4D), exhibited higher editing efficiency than WC3, which targeted only the D genome copy (Figure 1C). The relatively low editing efficiency of CP1, despite targeting all three homeologs, was attributed to sequence polymorphisms between the Bobwhite genome and the reference genome used for sgRNA design (Table S2).

The analysis of mutation patterns revealed distinct preferences among the sgRNAs: CP3 and WC3 predominantly induced insertions and deletions (InDels), whereas CP8 primarily generated single nucleotide mutations (Figure 1D, Figure S3). These findings suggest that sgRNAs targeting multiple homeologs simultaneously are more suitable for stable transformation applications, with CP3 being particularly effective due to its propensity to generate frameshift mutations in the coding sequence.

The protoplast-based evaluation system established here provides a rapid and reliable method for assessing sgRNA efficiency before stable transformation, enabling optimal target selection for subsequent wheat genome editing experiments.

### 2.2. Comparative Analysis of Transformation Methods for Generating Homozygous Mutants

Based on the protoplast screening results, we constructed a multi-target vector system for stable transformation. The pBUN411 backbone was modified to express four sgRNAs: CP1 and CP3 were driven by the *TaU6* promoter, whereas WC3 and CP8 were controlled by the *TaU3* promoter. These promoters were arranged in tandem to optimize expression efficiency. Two transformation strategies were evaluated: biolistic bombardment of the pBUN411-4 targets vector and the Agrobacterium-mediated co-transformation of pBUN411-CP3 and pBUN411-4 targets.

**Figure 2:**
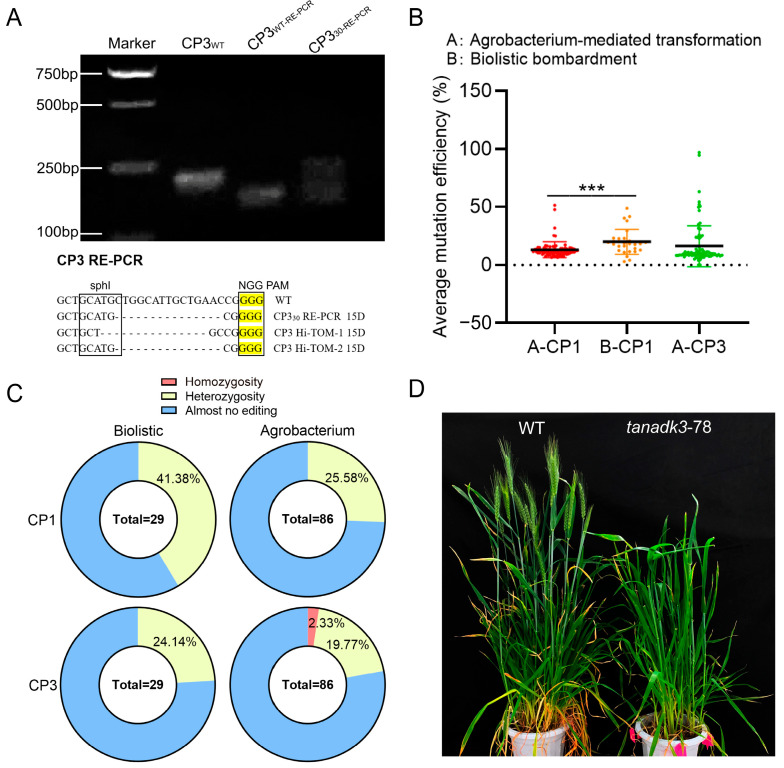
Statistics of stable transformation and gene editing efficiency. (**A**) PCR-RE gel electrophoresis of the CP3 target of Line No. 30. The type of gene editing detected by PCR-RE and Hi-TOM at the CP3 target of line No. 30 is also presented. (**B**) Hi-TOM detection of editing efficiency for different targets of Agrobacterium transformation and Biolistic bombardment (experimental results showing statistical significance. *** *p* < 0.001). (**C**) Statistics on the proportion of genotypes in tissues were obtained using two transformation methods. (**D**) Morphological records of the *TaNADK3* line at the booting stage (the homozygous mutation with double alleles was 2/86 or 2.33%).

The transformation experiments yielded 29 positive calli from biolistic bombardment and 86 transgenic lines from Agrobacterium-mediated transformation. Initial screening for mutations at the CP3 target site was performed using PCR restriction enzyme (PCR-RE) analysis, exploiting the *Sph*I restriction site within the target sequence. Analysis of line No. 30 revealed incomplete digestion of the PCR product, indicating the presence of mutations. Subsequent Sanger sequencing of the undigested 312 bp fragment confirmed the disruption of the *Sph*I restriction site (Figure 2A).

Deep sequencing analysis (Hi-TOM, 10,000 reads) of the CP1 and CP3 target sites revealed distinct mutation patterns between the two transformation methods. In T_0_ generation plants, biolistic bombardment produced significantly higher editing efficiency at the CP1 target compared to Agrobacterium transformation (Figure 2B), likely due to the introduction of multiple vector copies during bombardment. Using established criteria (Hi-TOM File S1), mutations were classified as significantly heterozygous (≥10%), homozygous (≥90%), or non-significant (<10%). At the CP1 target, biolistic bombardment generated heterozygous mutations in 41.38% of transformants, whereas Agrobacterium transformation was 25.58%. Notably, homozygous mutations were produced in 2.33% (2/86) of T_0_ plants only after co-transformation of the pBUN411-4 targets (containing CP3 target) with the pBUN411-CP3 vector using Agrobacterium transformation, demonstrating that repeated delivery of the same sgRNAs can enhance editing efficiency through an apparent additive effect.

The successful generation of homozygous *TaNADK3* mutants was confirmed through Hi-TOM sequencing of the CP1 and CP3 targets in T_1_ generation plants (14 lines). Phenotypic analysis revealed developmental delays in *TaNADK3* mutants [24], which reached the booting stage when wild-type Fielder wheat was already at the heading stage (Figure 2D). The mutants exhibited characteristic morphological alterations, including leaf shrinkage and yellow–green striping, with some plants failing to survive beyond the seedling stage. Biochemical analysis demonstrated significant reductions in both NADP(H) and chlorophyll content (Figure S2), confirming the successful functional knockout of *TaNADK3* across all three homeologs.

These results validate our protoplast-based screening approach and demonstrate the effectiveness of Agrobacterium-mediated transformation for generating homozygous wheat mutants, particularly when employing a co-transformation strategy with repeated sgRNA targets.

### 2.3. Impact of sgRNA Target Site Configuration on Gene Editing Efficiency

**Figure 3:**
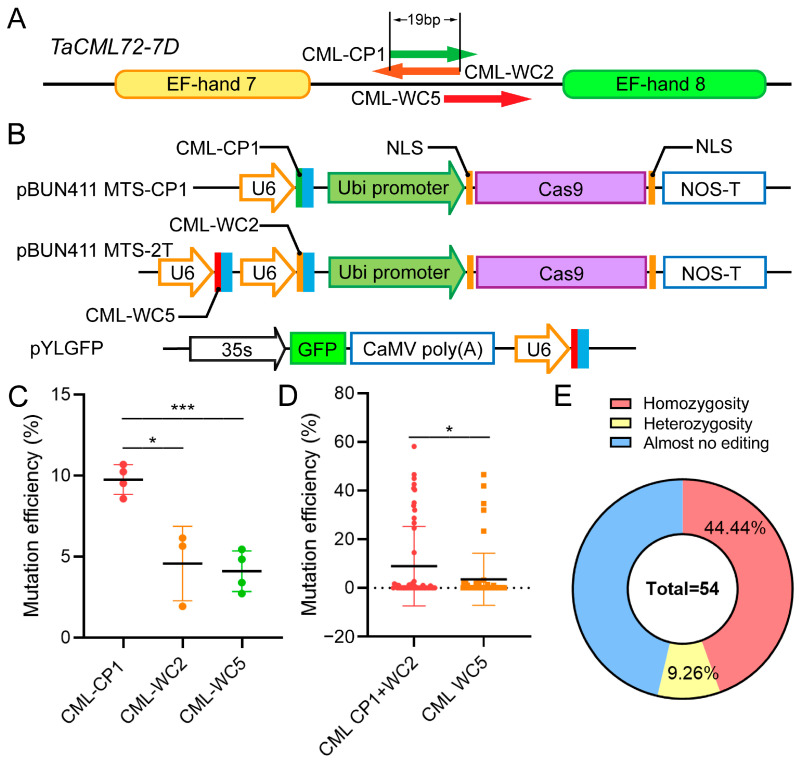
Results of the *TaCML72-7D* target gene knockout experiment. (**A**) Design of the *TaCML72-7D* target site. (**B**) Basic information about the stable transformation vector and the protoplast detection vector for *TaCML72-7D* knockout. (**C**) Verification results of gene editing based on protoplast transformation (experimental results showing statistical significance. * *p* < 0.05, *** *p* < 0.001). (**D**) Gene editing efficiency statistics for each target after stable transformation (experimental results showing statistical significance. * *p* < 0.05). (**E**) Gene editing efficiency statistics for *TaCML72-7D* (the homozygous mutation was 24/54 or 44.44%).

To investigate whether overlapping sgRNA targets could enhance editing efficiency similar to repeated targets, we conducted a systematic analysis using *TaCML72-7D* as a single-copy target gene. This experimental design eliminated the complexity associated with multiple homeologs present in the previously studied *TaNADK3* system. Three sgRNAs were designed for *TaCML72-7D*: CML-CP1, CML-WC2, and CML-WC5, with CML-CP1 and CML-WC2 sharing 19 bp of reverse-complementary sequence overlap (Figure 3A).

Initial validation of target efficiency was performed using protoplast transformation with individually constructed vectors pYLGFP-CML-CP1, pYLGFP-CML-WC2, and pYLGFP-CML-WC5 (Figure 3B). Deep sequencing analysis revealed significantly higher editing efficiency for the CML-CP1 target compared to both CML-WC2 and CML-WC5, and no significant difference was observed between the latter two targets (Figure 3C).

For stable transformation, two vectors were constructed: pBUN411-CML-2T, containing tandem CML-WC2 and CML-WC5 targets, and pBUN411-CML-CP1, carrying the CML-CP1 target sequence. Agrobacterium-mediated co-transformation of these vectors generated 54 regenerated plants. Due to the overlapping nature of CML-CP1 and CML-WC2 targets, and their partial overlap with CML-WC5, editing efficiency was analyzed at non-overlapping nucleotide positions. Contrary to expectations, the overlapping target configuration did not enhance mutation frequency, and no significant differences in editing efficiency were observed among the targets (Figure 3D). These results indicate that overlapping PAM sites may interfere with Cas9 protein binding or cleavage activity, suggesting that target site overlap should be avoided in sgRNA design.

After excluding non-specific amplification products from chromosome 7A during the Hi-TOM deep sequencing analysis, the mutations were classified using modified thresholds appropriate for single-copy genes: significantly heterozygous (≥8%), homozygous (≥20%), and non-significant edits (<8%). Under these criteria, 44.44% (24/54) of regenerated plants carried mutations in *TaCML72-7D* (Figure 3E), demonstrating substantial editing efficiency for single-copy wheat genes using this system.

To further evaluate the practicality of the gene editing system proposed in this study, we analyzed off-target occurrences. The CRISPR-P (http://crispr.hzau.edu.cn/CRISPR2/, accessed on 19 March 2023) website enabled the direct prediction of potential off-target sites for the *TaNADK3* and *TaCML72-7D* genes. Then, we detected only minimal levels of off-target editing (Table 1, Figure S4). These results indicate that this wheat gene editing system is suitable for the knockout of genes in a single chromosome (*TaCML72-7D*) or multiple chromosomes (*TaNADK3*). Thus, the system is safe in a predictable range.

### 2.4. Development of a BSMV-Mediated Gene Editing System for Wheat

Given the limitations of conventional Agrobacterium-mediated transformation, including platform specificity and patent restrictions, we explored the potential of Barley stripe mosaic virus (BSMV) as an alternative delivery system for the CRISPR components [25]. Initial validation of this approach utilized *TaPDS* as a reporter gene, as its knockout produces a readily observable photobleaching phenotype.

**Figure 4:**
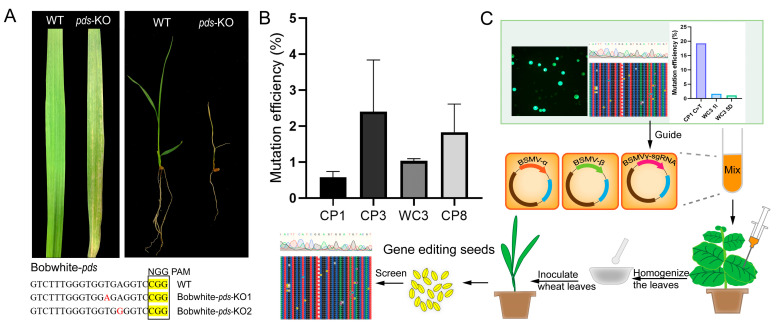
Creation and efficiency statistics of wheat knockout mutants based on BSMV delivery. (**A**) *TaPDS* knockout plants generated using VITF-edit (the left panel shows the streaking and bleaching produced after coating the leaves and the right panel shows the albinism of the next generation of wheat, mutations are shown in red). (**B**) Hi-TOM tests the editing efficiency of the VITF edit for different targets. (**C**) The improved process of VITF-edit-based gene editing technology for wheat.

The BSMV-based delivery system was constructed. The pCB301-BSMVsed delivery system consisted of three vectors. Following tobacco transformation with the mixed vectors, BSMV activity was confirmed via RT-PCR detection of BSMVγ tobacco transformation at 5–7 days post-transformation (Figure S3). Homogenized tissue from transformed tobacco leaves was subsequently used to inoculate wheat leaves. Characteristic phenotypes emerged approximately 15 days post-inoculation, including stripe whitening and disease symptoms (Figure 4A). Seeds collected from plants exhibiting albino leaves yielded two confirmed *TaPDS* knockout mutants, representing 20% editing efficiency. The mutation profiles were characterized using Hi-TOM sequencing at 1000 reads depth (Figure 4A).

To further validate the BSMV delivery system, we constructed four vectors (pCB301-BSMV-CP1, pCB301-BSMV-CP3, pCB301-BSMV-WC3, and pCB301-BSMV-CP8) targeting *TaNADK3* using previously designed sgRNA. After transforming Bobwhite-Cas9^+^ wheat using the BSMV delivery system, the Hi-TOM analysis revealed differential editing efficiencies among the targets. CP3 achieved the highest efficiency (2.5%), followed by CP8 (1.8%) and WC3 (1.08%) (Figure 4B). Notably, the relative efficiency of these targets mirrored the patterns observed in protoplast experiments, suggesting that protoplast-based screening can effectively predict BSMV-mediated editing outcomes.

Based on these findings, we proposed an optimized strategy for BSMV-mediated wheat gene editing (Figure 4C). This approach incorporates the initial protoplast-based validation of sgRNA efficiency, followed by the construction of BSMV vectors carrying the most effective sgRNAs for viral delivery. This streamlined protocol provides an accessible alternative to conventional transformation methods for wheat genome editing in standard laboratory settings.

## 3. Discussion

This study addresses critical challenges in wheat genome editing by developing and validating an integrated approach that combines protoplast-based sgRNA screening, optimized transformation strategies, and a novel viral delivery system. Our research findings provide some substantial insights into the wheat genome editing field and offer practical solutions for laboratories with limited resources.

### 3.1. Protoplast-Based Evaluation System Enhances sgRNA Design Efficiency

The establishment of a protoplast-based screening system represents a significant advancement in sgRNA design optimization. By enabling the rapid assessment of editing efficiency before stable transformation, this system substantially reduces the time and resources required for successful wheat genome editing. Our results demonstrate that vector size significantly influences transformation efficiency in wheat protoplasts, with smaller vectors showing superior performance. This finding addresses the particular challenges posed by the fragile nature of wheat protoplasts [26] and provides practical guidelines for vector design.

### 3.2. Multiple Factors Influence sgRNA Editing Efficiency

Our systematic analysis of sgRNA performance revealed the complex determinants of editing efficiency. Although the CP1 target consistently showed low editing efficiency across multiple protoplast experiments, this cannot be attributed solely to the single nucleotide polymorphism (SNP) between Bobwhite and Fielder wheat varieties. Previous studies have demonstrated that not all SNPs significantly impact editing efficiency [27,28]. However, the position of mismatches within the sgRNA spacer region, particularly in the seed sequence, can critically affect targeting efficiency [29]. Beyond sequence complementarity, our results suggest that intrinsic factors, such as sgRNA secondary structure and chromatin accessibility in the target region, may substantially influence editing outcomes. This observation emphasizes the importance of considering both sequence-specific and structural features in sgRNA design, particularly for complex polyploid genomes such as wheat. The consistently low efficiency of CP1 across multiple experiments suggests that inherent structural or accessibility factors, rather than sequence variations alone, may be limiting its effectiveness.

### 3.3. Optimized Transformation Strategies for Generating Homozygous Mutants

The comparative analysis of transformation methods revealed distinct advantages and limitations of biolistic bombardment versus Agrobacterium-mediated transformation. Although biolistic bombardment achieved higher initial editing efficiency in T_0_ plants, likely due to the delivery of multiple vector copies, Agrobacterium-mediated transformation proved more effective for obtaining homozygous mutants. This observation aligns with previous studies suggesting that the sustained expression of CRISPR components through Agrobacterium transformation enhances mutation frequency over multiple generations [30].

A particularly novel finding was that co-transformation with vectors carrying the same sgRNA significantly increased the copy number and half-life of sgRNA, thereby enhancing the editing efficiency at the target sites [31,32,33]. This strategy resulted in a 2.33% homozygous mutation rate in T_0_ plants for the multi-copy *TaNADK3* gene, representing a significant improvement over conventional single-vector approaches. When transforming with only a single vector, the expression of the same sgRNA target driven by different small nuclear RNA promoters can be tandemly expressed to increase the expression level of sgRNA, thereby improving editing efficiency [20]. Moreover, our subsequent investigation revealed that overlapping but non-identical sgRNA targets can interfere with editing efficiency, providing important guidance for future sgRNA design strategies.

### 3.4. BSMV-Mediated Delivery System Offers an Accessible Alternative

The development of a BSMV-based delivery system addresses the accessibility limitations of conventional transformation methods. Our results demonstrate that this approach can achieve editing efficiencies of up to 20% for single-copy genes, making it a viable alternative for laboratories without access to specialized transformation platforms. Importantly, the correlation between protoplast-based screening results and BSMV-mediated editing efficiency provides a reliable predictive tool for sgRNA selection.

The system’s effectiveness was validated through successful editing of both single-copy (*TaCML72-7D*) and multi-copy (*TaNADK3*) genes, with minimal off-target effects. The phenotypic and biochemical characterization of *TaNADK3* mutants [24], showing reduced NADP(H) and chlorophyll content, confirms the system’s ability to generate relevant gene mutations.

### 3.5. Future Perspectives and Limitations

Although our integrated approach significantly improved the accessibility and efficiency of wheat genome editing, several aspects warrant further investigation. The mechanism underlying the interference between overlapping sgRNA targets is yet to be fully elucidated. Additionally, the optimization of sgRNA design algorithms, specifically for wheat’s hexaploid genome, could further enhance editing efficiency. The BSMV-mediated system, although promising, currently shows lower editing efficiency compared to conventional transformation methods. Future optimization of viral vectors and delivery protocols may help bridge this efficiency gap. Nevertheless, the system’s accessibility and the ability to rapidly screen multiple sgRNAs make it a valuable addition to the wheat genome editing toolkit.

In conclusion, this study provides a comprehensive framework for efficient wheat genome editing, combining protoplast-based screening, optimized transformation strategies, and an accessible viral delivery system. These advances significantly reduce the technical and resource barriers to wheat genome editing, enabling broader application of this technology in both basic research and crop improvement programs.

## 4. Materials and Methods

### 4.1. Plant Materials, Plasmids, and Bacteria Strains

A marker-free transgenic wheat (*Triticum aestivum*) Fielder was used for transformation in this study. Fielder and tobacco (*Nicotiana tabacum*) were acquired from the Crop Germplasm Bank of China. Bobwhite wheat was provided by Prof. Yanpeng Wang (Institute of Genetics and Developmental Biology, Chinese Academy of Sciences). All plants were grown in 8″ pots containing V9 seedling substrate (Shouguang City Tianfeng horticultural materials factory). There were 8 plants per pot at 25 °C, 15L:9D with 200 μmol·m^–2^·s^−1^ light intensity. When the season was right, some wheat was transplanted to the field.

Vectors containing SpCas9 (pYLCRISPR-Cas9P35s-H) and OsU6-sgRNA scaffold (pYLsgRNA-OsU6a/LacZ) were kindly provided by Prof. Yaoguang Liu (Southern China Agricultural University) [34]. The pBUN411 gene editing vector was provided by Xiaojie Wang (Northwest A&F University) [35]. The VITF-edit gene editing system, based on BSMV, was provided by Prof. Yanpeng Wang (Institute of Genetics and Developmental Biology, Chinese Academy of Sciences) [25]. The vectors containing EGFP (pTF486 and pGL486) were provided by Prof. Fei Yu (Northwest A&F University). *Agrobacterium* and Top10 *E. coli* strains were obtained from laboratory stocks.

### 4.2. Construction of Plasmids

pYLsgRNA-OsU3/LacZ was used as the vector backbone [34]. The *TaU6* (GenBank accession number X63066.1) and *TaU3* (X63065.1) promoters were cloned from the Fielder genome. After reconstruction, pYLsgRNA-TaU6/LacZ and pYLsgRNA-TaU3/LacZ were obtained. The pBUN411 vector was modified to be identical to the pYLCRISPR-Cas9P35s-H series vector adaptor at the sticky end of the BsaI restriction site [35]. Four targets of CP1, CP3, WC3, and CP8 were designed for the *TaNADK3* knockout, whereas CML-CP1, CML-WC5, and CML-WC2 were designed for the *TaCML72-7D* knockout. CP1 and CP3 were driven by *TaU6* promoters, whereas WC3 and CP8 were driven by *TaU3* promoters. The sgRNAs for *TaCML72-7D* were all driven by the *TaU6* promoter. The assembly target was based on the method of Golden Gate cloning [36].

Then, the protoplast expression vector of pYLGFP wheat was constructed. The fusion expression vector PGL486-Cas9 was constructed from the Ubi-Cas9 expression cassettes on the pBUN411 vector and the pGL486 vector so that the Cas9 protein could carry the GFP tag. In addition, a 35S-GFP-CaMV poly (A) signal GFP expression cassette was inserted into the NdeI (968) site of pYLsgRNA-TaU6/LacZ to indicate the conversion efficiency of protoplasts. The vector was named pYLGFP-sgR. The sgRNA in the vectors used for protoplast transformation were all driven by the *TaU6* promoter, and the method was the same as above.

The vector construction methods for the VITF-edit system followed those described by Li et al. [25].

### 4.3. Isolation and Transformation of Wheat Protoplasts

Before the isolation of protoplasts, the seedlings of wheat cultivar Fielder/Bobwhite were grown under a 15L:9D dark cycle at 25 °C in a growth room for 7–12 days. The preparation of the protoplasmic system followed Shan et al. [37], with some modifications.

Healthy growing wheat leaves were cut into fine strips transversely and digested with an enzyme solution (1% Cellulase R10, 0.2% Macerozyme R10, 0.6 M mannitol, 10 mM KCl, 20 mM MES pH5.7, 10 mM CaCl_2_, 0.1% BSA, and 0.035% β-Mercaptoethanol), followed by vacuum-infiltration for 15 min using a vacuum pump at −15~−20 (in Hg). The vacuum-treated wheat leaves were placed in incubators at 25 °C for digestion at 40 rpm for 90~120 min. Protoplasts were isolated via filtration through two layers of 70 μm nylon meshes into round bottom tubes. The enzymolysis reaction was terminated by adding an equal volume of W5 solution (154 mM NaCl, 125 mM CaCl_2_·2H_2_O, 5 mM KCl, and 2 mM MES pH5.7). Pellets were collected at 30 g, 10 min. The supernatant was removed and the protoplast precipitation was washed twice with W5 solution. A total of 10~20 mL W5 solution was added to suspend the protoplasts, and the protoplasts were placed on ice for 40 min. The supernatant was then discarded and re-suspended in MMG solution (0.4 M mannitol, 15 mM MgCl_2_, and 4 mM MES pH5.7).

The protoplast transformation was carried out in PEG solution [40% (*w*/*v*) PEG 4000, 0.2 M mannitol, and 100 mM CaCl_2_]. The transformation mixtures (10 µg pYLGFP-sgR plasmid with 100 µL protoplasts in 110 µL PEG solution) were agitated gently. After 25 min at room temperature in the dark, the protoplasts were harvested and washed with 500 µL W5 solution. They were then centrifuged (100 g for 5 min) and resuspended in 300 µL W5 solution and cultured in 96-well plates in the dark at 25 °C. Fluorescence was detected after 16~18 h of culturing and the protoplasts were collected at 48 h for Hi-TOM sequencing. The conversion efficiency of 60~80% was determined using fluorescence detection. Every 10 reactions constituted the minimum amount of detection.

### 4.4. Primers and Guide RNA Design

Primers were designed using NCBI (https://www.ncbi.nlm.nih.gov/tools/primer-blast/, accessed on 7 January 2021) and Primer Premier 5. sgRNAs targeting *TaNADK3* and *TaCML72-7D* were designed by CRISPR-Cereal (http://crispr.hzau.edu.cn/CRISPR-Cereal/index.php accessed on 13 January 2021) and WheatCrispr (https://crispr.bioinfo.nrc.ca/WheatCrispr/, accessed on 15 January 2021).

### 4.5. Wheat Transformation

The vector transformation based on *Agrobacterium Tumefaciens* in this experiment was provided by the National Key Laboratory of Crop Improvement for Stress Tolerance and Production, NWAFU, referring to the experiment conducted by Wang et al. [18]. The plasmid transformation based on biolistic bombardment was primarily based on the work of Ishida et al. [38].

### 4.6. Detection Analysis of Edited Mutations

Genomic DNA was extracted from candidate T_0_ transgenic mutant plants using the CTAB method. A PCR-restriction enzyme (PCR-RE) assay was performed for these genes. The *TaNADK3* fragments with restriction sites were amplified. The target bands were recovered using the gel recovery kit (Genestar, Beijing, China) and were digested with SphI (Promaga, Madison, WI, USA) for 4 h. The digestion products were observed using 2% agarose gel electrophoresis. The uncut strip was recovered and attached to pMD19-T (TaKaRa, Osaka, Japan). Five monoclonal clones were randomly selected for each transformation for Sanger sequencing. Hi-TOM (http://121.40.237.174/Hi-TOM/login.php, accessed on 27 April 2023) was used for the second-generation sequencing of all wheat mutants [39,40]. Protoplast transformation is usually sequenced at a depth of 10,000 reads. Stable transformation events are usually sequenced at a depth of 1000 reads, which can accurately determine the type and mutation efficiency of wheat gene editing.

### 4.7. Phenotypic Detection of TaNADK3-Edited Mutations

Fielder plants with sequenced homozygous genotypes (*TaNADK3*, *4A*, *4B*, *4D*) were planted with seeds in 8″ pots. A total of 6~8 wheat plants were planted in each pot, and 3 replicates were planted in mutant and wild-type wheat. The state of both was recorded during growth. When the mutant grew to the jointing stage, together with wild-type leaves under the same growth tendency, chlorophyll and NADP(H) were measured following the method described by Noctor et al. [41].

## 5. Conclusions

In conclusion, this study optimized the selection of sgRNAs for wheat gene editing through protoplast transformation, thereby enhancing the efficiency of the CRISPR-Cas9 system. We found that repeated transformation of the same sgRNA target can improve editing efficiency at that locus, while the use of sgRNAs with overlapping spacer regions leads to reduced editing efficiency. Additionally, the workflow we established was successfully applied to the BSMV-based wheat gene editing transformation system, demonstrating good versatility. This research provides a practical solution for conventional laboratories with limited resources to conduct efficient wheat gene editing, and is expected to accelerate the research in wheat functional genomics and molecular breeding.

## Figures and Tables

**Table 1:** Prediction of possible off-targets.

Target Gene	Target Site	Off-Target Site Prediction	Off-Target
*TaNADK3*	CP1	TraesCS1B02G376800	1.04%
*TaNADK3*	CP3	TraesCS1B02G440300	No
*TaCML72-7D*	CML-CP1	XM_044587532.1	2.09%
*TaCML72-7D*	CML-WC2	XM_044566133.1	No
*TaCML72-7D*	CML-WC5	TraesCS1D02G312500	No

## Data Availability

The original contributions of the study are included in the article; further inquiries can be directly addressed to the corresponding author.

## Supplementary Materials

The following supporting information can be downloaded at: https://www.mdpi.com/article/10.3390/ijms26083796/s1.
