# Supplementary material for: An Optimized Editing Approach for Wheat Genes by Improving sgRNA Design and Transformation Strategies
Source: Int J Mol Sci. 2025 Apr 17;26(8):3796. doi: 10.3390/ijms26083796 (PMC12028029; doi:10.3390/ijms26083796)
Supplement: Supplementary file 1 [file ijms-26-03796-s001.zip › ijms-3521313-supplementary.pdf]

Hi-TOM Supplementary Data
We have provided the raw data from the Hi-TOM sequencing in the attached files.
1. Data of *PDS* gene editing site in tobacco (USR-9053)
2. Data of protoplast gene editing (USR-9345)
3. Data of Off-target gene detection (USR-9731)
4. Data of target site in T1 wheat plants (USR-10473)
5. Data of target site in callus created by *Agrobacterium*-mediated transformation(USR-10585)
6. Data of target site in BSMV-acting gene editing mutants (USR-13214)
7. Data of sgRNA target sites' difference between wheat Chinese spring and Bobwhite (USR-11247)
8. Data of Off-target gene detection-2 (USR-13545)
9. Data of supplementary experiments for protoplast gene editing (USR-30302), (USR-30606), (USR-30607), (USR-30608), (USR-30805), (USR-30806), (USR-30807)

Supplementary Table S1. In Fielder wheat, Gene IDs, chromosome location, and sgRNA sequence information of the target gene

Target gene	Gene ID	Chromosome	Target sequence	Target genome	Target Name
TaNADK3	TraesCS4A02G221400<br>TraesCSU02G067800<br>TraesCS4D02G095400	4A<br>4B<br>4D	CTATGATGCGGGTTTCGCAAGGG	ABD	CP1
			ATGCTGGCATTGCTGAACCGGGG	ABD	CP3
			ACTGGAGACTAGTCCCCTTAAGG	D	WC3
			GCTGCAGAATTCTCACACAAGGG	AD	CP8
TaCML72	TraesCS7D02G507100	7D	CGTCAAGCGAAGGTGCCTGAGGG	7D	CML-CP1
			CAGGCACCTTCGCTTGACGTCGG	7D	CML-WC2
			GGTGCCTGAGGGAGGCATTCGGG	7D	CML-WC5
TaPDS	TraesCS4A02G004900T<br>raesCS4B02G300100<br>TraesCS4D02G299000	4A<br>4B<br>4D	GTCTTTGGGTGGTGAGGTCCGG	ABD	B-PDS

Note: The PAM sites are highlighted in red.

Supplementary Table S2. Sequence differences between Fielder and Bobwhite at CP1 targets

Chromosome	Sequence near CP1 target
Fielder 4A	AAGAATGCCTGCTATGATGCGGGTTTCGCAAGGGTTGATGGTCAT
Fielder 4B	AAGAATGCCTGCTATGATGCGGGTTTCGCAAGGGTTGATGGTCAT
Fielder 4D	AAGAATGCCTGCTATGATGCGGGTTTCGCAAGGGTTGATGGTCAT
Bobwhite 4A	AAGAATGCCTGCTATGATGCGGGTTTCGCAAGGGTTGATGGTCAT
Bobwhite 4B	AAGAATGCCTGTTATGATGCGGGTTTCGCAAGGGTTGATGGTCAT
Bobwhite 4D	AAGAATGCCTGCTATGATGCGGGTTTCGCAAGGGTTGATGGTCAT

Note: The gRNA sites are highlighted in blue, the differences between gRNA sites of different chromosomes are highlighted in red, the PAM sites are highlighted in yellow background.

Supplementary Table S3. Main mutation types data generated by different targets in protoplast experiments

Target Name	Sequence Information	
CP1	GGTTTCGCAAGGGTTGATGGTCATCCCTGTCCAACATTGTTTGCAAATTGGTT	WT
	GGTTTCGCAAGGGTTGATGGTCATCCCTGTCCAACATTGTTTGCAAATCGGTT	SNP
CP3	GGTTGCTGCTGTGCTTGGCTGCATGCTGGCATTGCTGAACCGGGGTAGGAC	WT
	GGTTGCTGCTGTGCTTGGCTGCTG------------------------------CCGGGGTAGGAC	15D
	GGTTGCTGCTGTGCTTGGCTGCATGCTGGCATTGCTGA--CCGGGGTAGGAC	1D
	GGTTGCTGCTGTGCTTGGCTGCATGCTGGCATTGCTGAAACCGGGGTAGGAC	1I
	GGTTGCTGCTGTGCTTGGCTGCATGCTGGCATTGC--------CCGGGGTAGGAC	4D
	GGTTGCTGCTGTGCTTGGCTGCATGCTGGCATTGCTGAATCCGGGGTAGGAC	1I
WC3	ACTGGAGACCAGTCCCCTTAAGGCACAGTTTCCTACTTGCAATGTTTTCTCT	WT-4A
	ACTGGAGACCAGTCCCCCTTAAGGCACAGTTTCCTACTTGCAATGTTTTCTCT	1I
	ACTGGAGACCAGTCCCCTTAAGGCACAGTTTCCTACTTGCAATGTTTCCTCT	SNP
	ACTGGAGACCAGTCCTCTCAAGGCACAGTTTCCTACTTGCAATGTCTTCTCT	WT-4B
	ACTGGAGACTAGTCC----------AGGCACAGTTTCCTACTTGCAATGTTTTCTCT	5D,SNP
CP8	TGGGGCACATTCTTTGCTGCAGAATTCTCACACAAGGGACTTATCCCAGCTG	WT-4D
	TGGGGCACATTCTTTGCTGCAGAATTCTCGCACAAGGGACTTATCCCAGCTG	SNP
	GTGGAAACTTTGTTGCTTGCTTGATTGCATCC	Large Indel
	TGGGGCACATTCTTTGTTGCAGAATTCTCACACAAGGGACTTATCCCAGCTG	SNP
	TGGGGCACATTCTTTGCTGCAGAATTCTCACACAAGGGGCTTATCCCAGCTG	SNP
	TGGGGCACATTCTTTGCTGCAGAATTCTCACACAAGGAACTTATCCCAGCTG	SNP
	TGGGGCACATTCTTTGCTGCAGAATTCTCACACAAGGGACTTATCCCTGCTG	SNP

Note: The PAM sites are highlighted in yellow background.

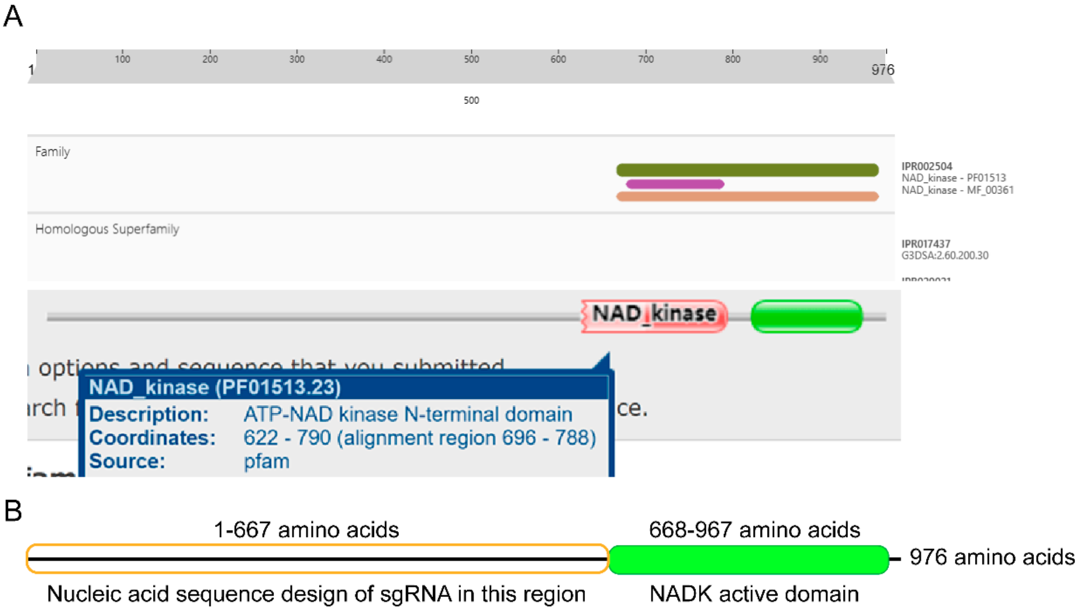

Supplementary Figure S1. Wheat NADK3 protein sequence analysis and sgRNA design region selection.

(A) Distribution of the active structural domains of NADK3 protein obtained using InterPro and Pfam analyses, respectively. Detailed analysis results are available on the webpage(https://www.ebi.ac.uk:443/interpro//result/InterProScan/iprscan5-R20230219-013156-0592-86093009-p1m/). (B) Schematic diagram of the sgRNA design region.

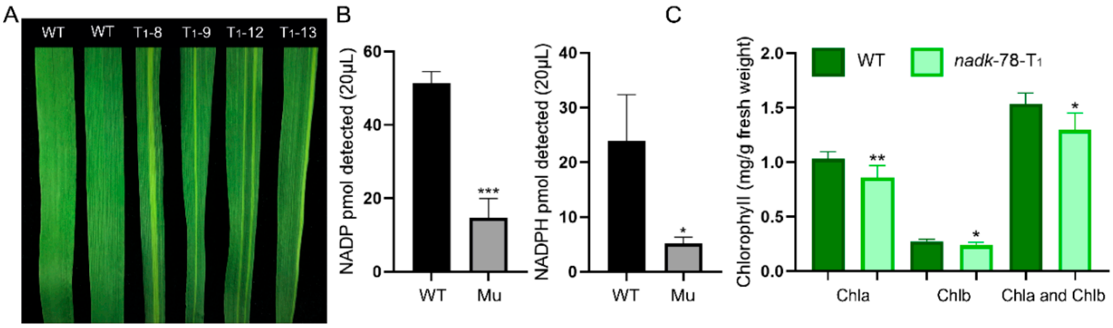

Supplementary Figure S2. Phenotypic and physiological measurements of *tanadk3 4A*, *4B*, *4D*.

(A) Leaf phenotype of *tanadk3* line at booting stage. (B) Determination of NADP(H) content of *tanadk3* line, 3 replicates per group. (C) Determination of Chlorophyll content of *tanadk3* line, 6 replicates per group.

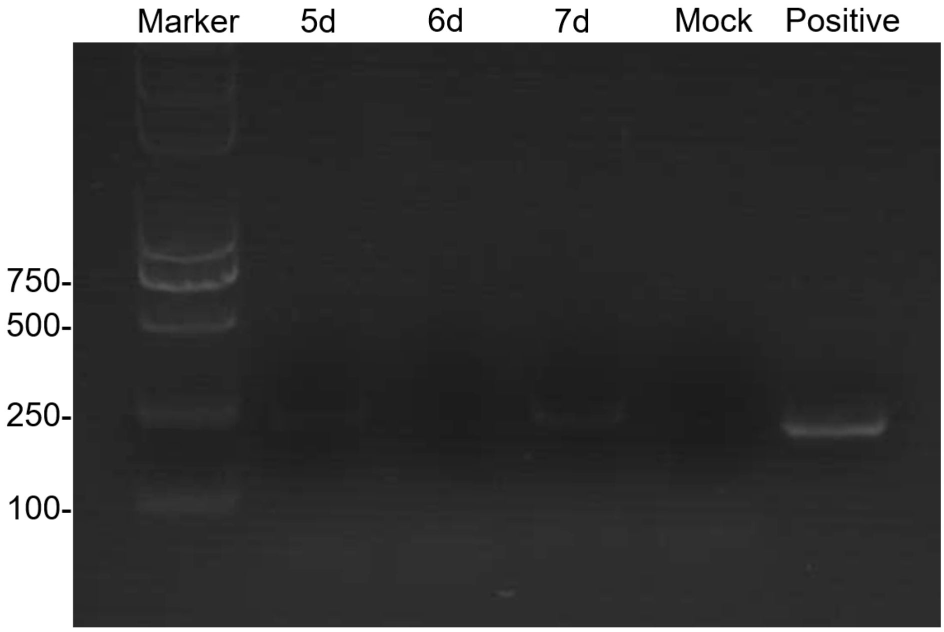

Supplementary Figure S3. Agarose gel electrophoresis plots of tobacco leaf RNA after reverse transcription using PCR to detect BSMVγ light chain.

***TaNADK3* CP1 off-target**

GTATGATGCTGCTGTCGCAATGGCTGA WT
GTATGATGCCGCTGTCGCAATGGCTGA off-target

***TaCML72-7D* CP1 off-target**

GCCGACGTCAAGCGAAGGTGCCTGAGGGAGGC WT
GCCGACGTCAAGCGAAGGTG-CTGAGGGAGGC off-target

Supplementary Figure S4. Base sequence changes in off-target editing.

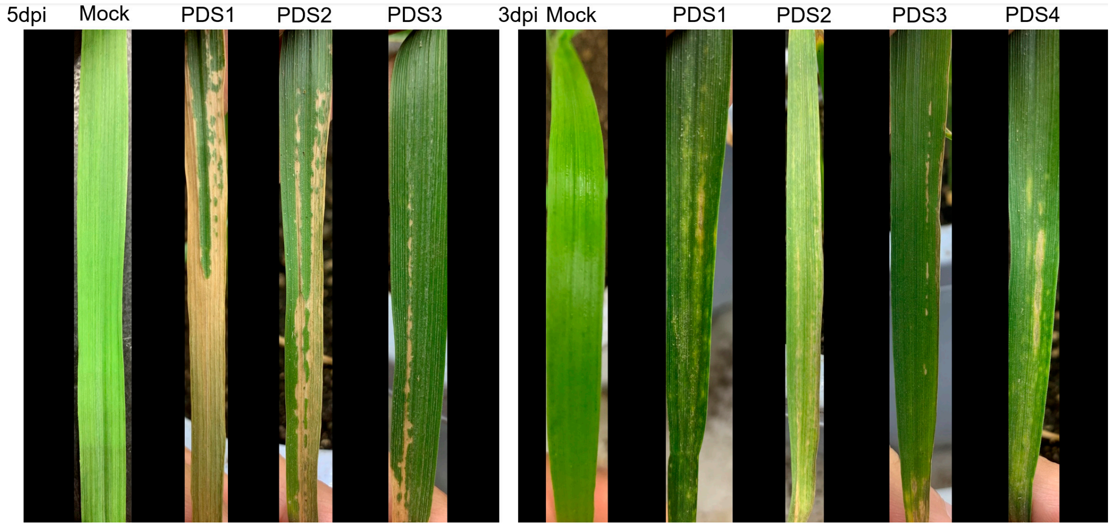

Supplementary Figure S5. Leaf phenotype of wheat PDS knockout mutants delivered by BSMV
